# Notoginsenoside Fe suppresses diet induced obesity and activates paraventricular hypothalamic neurons†

**DOI:** 10.1039/c8ra07842d

**Published:** 2019-01-11

**Authors:** Hongli Li, Yalei Liu, Chuhe Liu, Lingling Luo, Yin Yao, Fei Li, Liufang Yin, Lai Xu, Qingchun Tong, Cheng Huang, Shengjie Fan

**Affiliations:** Drug Discovery Laboratory, School of Pharmacy, Shanghai University of Traditional Chinese Medicine Shanghai 201203 China shengjiefan@shutcm.edu.cn; Brown Foundation Institute of Molecular Medicine and Program in Neuroscience, Graduate School of Biological Sciences, University of Texas McGovern Medical School Houston TX USA

## Abstract

Obesity has become a major public health challenge worldwide. Energy imbalance between calorie acquisition and consumption is the fundamental cause of obesity. Notoginsenoside Fe is a naturally occurring compound in *Panax notoginseng*, a herb used in the treatment of cardiovascular diseases in traditional Chinese medicine. Here, we evaluated the effect of notoginsenoside Fe on obesity development induced by high-fat diet in C57BL/6 mice. Our results demonstrated that notoginsenoside Fe decreased food intake and body weight, as well as protected liver structure integrity and normal function. Metabolic cage analysis showed that notoginsenoside Fe also promoted resting metabolic rate. In addition, intracerebroventricular (i.c.v) injection of notoginsenoside Fe induced C-Fos expression in the paraventricular nucleus (PVH) but not the arcuate nucleus (ARC) of the hypothalamus. These results suggest that Fe may reduce body weight through the activation of energy-sensing neurons in the hypothalamus.

## Abstract 2

Notoginsenoside Fe, a naturally occurring compound in *Panax notoginseng*, significantly reduces body weight, promotes metabolic rate, and suppresses food intake through activating C-Fos expression in PVH in high-fat diet induced obese mice.

## Graphical abstract

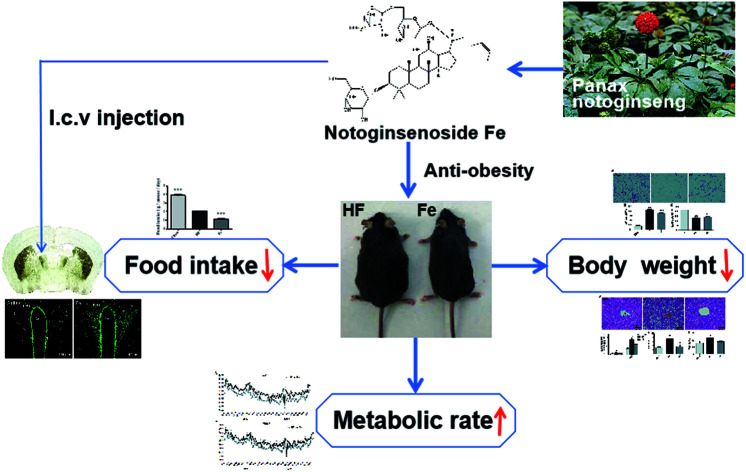

## Introduction

Obesity is defined as abnormal or excessive fat accumulation which is harmful to human health by the World Health Organization (WHO). Obesity is a major risk factor for cardiovascular diseases, type 2 diabetes mellitus, hyperlipidemia, hypertension, fatty liver disease and cancer.^1,2^ A common feature of most obese individuals is overconsumption of food, resulting in energy imbalance between calorie intake and consumption.^3,4^ Thus, suppression of feeding has been one of the major focuses of anti-obesity pharmacotherapy investigation for decades. However, most commercial anti-obesity drugs have limited efficacy and are hampered by their serious side effects. Thus, there is an urgent need to improve the development of anti-obesity drugs with better safety and potent efficiency.

Consistently, the hypothalamus plays a crucial role in controlling food intake and energy expenditure. The ARC contains receptors for various hormones modulating energy balance, such as leptin, insulin, ghrelin and others.^5^ The PVH as the primary hypothalamic site receives a signal output from the ARC and projects it to brain satiety regions, PVH lesions result in hyperphagic obesity, while neuron activity in the PVH acutely increases energy expenditure and diminishes energy intake, suggesting that PVH plays an indispensable role in regulating energy balance.^6–9^

*Panax notoginseng* is a well-known traditional Chinese medicine in the genus *Panax*, belonging to the Araliaceae family,^10^ and has been gaining popularity in the West recently. *Panax notoginseng* has been used in Chinese medicine for dissolving stasis, stopping bleeding, alleviating swelling, pain, hemoptysis, hematemesis, blood in stools, uterine bleeding, traumatic bleeding, abdominal irritation, and joint swelling.^11^ Ginsenosides, triterpene saponins, are the main bioactive ingredients in *Panax notoginseng*.^12,13^ Many studies have confirmed that ginsenosides have multiple pharmacological activities, such as protecting myocardial and cerebral ischemia-reperfusion injury,^14,15^ lung injury,^16^ diabetic encephalopathy,^17^ neurons injury,^18,19^ preventing restenosis after percutaneous transluminal angioplasty^20^ and cervical cancer.^21^

Notoginsenoside Fe is a saponin found in *Panax notoginseng* leaves.^22^ The pharmacological effect of notoginsenoside Fe has not been studied yet. In present study, we found that notoginsenoside Fe reduced food intake and body weight, lowered fasting blood glucose, and increased energy expenditure in high-fat-induced obese mice. Furthermore, notoginsenoside Fe inhibited feeding in diet-induced obese (DIO) mice with i.c.v injection.

## Materials and methods

### Animals and treatments

Male C57BL/6 mice, 6 weeks old, were purchased from Shanghai Laboratory Animal Center of Chinese Academy of Science (Shanghai, China). All animals were housed under a 12 h light/dark cycle at controlled temperature (22 ± 1 °C) with free access to food and water. All animal experiments used in this study were complied with the Institutional Animal Care guidelines approved by the Experimental Animal Ethical Committee (SZY201707004, Shanghai, China) at Shanghai University of Traditional Chinese Medicine.

After one week adaptive feeding, these mice were divided into two groups with a low-calorie diet as the chow control (10% of calories derived from fat, Research Diets, New Brunswick, NJ, D12450B) and a high-fat (HF) diet group (60% of calories derived from fat, Research Diets, New Brunswick, NJ, D12492). After 3 month-HF induction, obese mice were randomly divided into HF group and notoginsenoside Fe group. Mice in notoginsenoside Fe group were intraperitoneal (i.p) injected with notoginsenoside Fe (10 mg kg^−1^ d^−1^, dissolved in saline with 10% DMSO, purchased from PUSH BioTech, Chengdu, China), along with chow and HF group mice being injected the same volume of saline with 10% DMSO as control and the treatment lasted 2 weeks.

Chow diet-fed mice were randomly divided into two groups and i.p injected with saline (with 10% DMSO) or notoginsenoside Fe (10 mg kg^−1^ d^−1^, dissolved in saline with 10% DMSO) for 6 days. Body weight and food intake were recorded every day throughout the experiment.

### Intraperitoneal glucose tolerance test (IPGTT)

After the 2 week-treatment, mice were fasted over-night. Fasting blood glucose (0 min) was measured as the baseline of glucose values through tail vein before the injection of glucose intraperitoneally (1 g kg^−1^ body weight). Additional blood samples were measured at regular intervals (15, 30, 60, 90, 120 min) during the glucose tolerance test.

### Metabolic cage measurements

After the 2 week-treatment, fat and lean mass were measured by EchoMRI™ (United Well Technologies Limited, China), to analyze the fat/body weight and lean/body weight. The mice were placed into individual metabolic chambers of Columbus Instruments Comprehensive Lab Animal Monitoring System (CLAMS, Columbus Instrument, USA). Metabolic data were collected for continuous 6 days, including oxygen consumption (VO_2_), carbon dioxide generation (VCO_2_), and respiratory exchange ratio (RER). Saline or notoginsenoside Fe was i.p injected around 12 p.m. every day. Mice had free access to HF-diet and water.

### Stereotaxic surgery

Stereotaxic surgeries for drug delivery to brain was performed as previously described.^23^ In brief, DIO mice were anesthetized with inhaling isopentane (RWD Life Science Co., China) and placed in a stereotaxic frame (RWD Life Science Co., China). Guide cannula (26 gauge, 5 mm, RWD Life Science Co., China) were inserted into right lateral brain ventricle with a coordinate (anterior–posterior: 0.3 mm, midline-right: 1 mm, dorsal–ventral: 2.5 mm). After a 7 day-recovery, mice were tested with angiotensin (Tocris a biotechne brand, China) to confirm surgery would be successful (an intensive drinking behavior immediately).

### Intracerebroventricular injection (i.c.v) and food intake measurement

For experiments, mice with guide cannula randomly divided into two groups. After over-night fasting, saline (2 μl per mouse) or notoginsenoside Fe (5 μM in 2 μl per mouse) was slowly administered through the guide cannula, the injection volumes and flow rate as previously described.^24–26^ After injection, mice were re-fed and food intake amount was measured for time periods of 0.5, 1, 2, 4, 6 h, respectively.

### Immunohistofluorescence staining

After 1 h of saline and notoginsenoside Fe administration mice was anesthetized with 20% ethyl carbamate, then the brain was fixated with 10% formalin through cardiac perfusion. Immunohistofluorescence staining was performed as previously described.^23^ In brief, the sections sequentially were distributed into 4 wells of 24 well plate filled with PBS containing 0.1% sodium azide. Brain sections were incubated with rabbit anti-C-Fos (1:1500, Millipore) and visualized with Alexa fluor® 488-conjugated affinipure goat anti-rabbit IgG (1:400, Jackson immunoresearch).

C-Fos expression was determined as previously described.^23^ Briefly, three matched brain sections containing PVH or ARC were chosen from each mouse, respectively. All immune-positive neurons numbers in the PVH or ARC with clear profile were counted and the average of C-Fos neuron number throughout three sections was used as a representative number of C-Fos positive neurons in each mouse.

### Tissue collection

At the end of animal experiment study, mice were fasted over-night and anesthetized with 20% ethyl carbamate (SCR, Sinopharm Chemical Reagent Co. Ltd, China) dissolved in saline and cardiac blood was taken. The liver and white adipose tissue (WAT) were frozen rapidly in liquid nitrogen and stored at −80 °C for the following experiments. Meanwhile, a part of liver and WAT were fixed in 10% formalin for morphology analysis.

### Histopathological assessment

The liver and WAT fixed in 10% formalin were embedded in paraffin and sectioned in 5 μm, and stained with hematoxylin–eosin.

### Hepatic lipid levels measurement

Frozen liver tissue of 50 mg were homogenized in 0.5 ml lysis buffer according to our previous protocol (1 ml 1 M Tris–HCl, pH 8.0; 7 ml 1 M NaCl; 0.5 ml Triton X-100, diluted with ddH_2_O to 50 ml).^27^ After that, the homogenate were mixed with equal volume of chloroform through 20 s vortex. The supernatant solution and protein layer were removed after centrifuging at 4 °C and 13 200 rpm for 10 min, the lower chloroform layer was dried and re-suspended with 50 μl isopropyl alcohol to measure the total cholesterol (TC) and triglyceride (TG) contents with assay kit (Dongou, Zhejiang, China).

### Real-time polymerase chain reaction (RT-PCR) analysis

Total RNA was extracted from 80 mg liver tissues using 800 μl RNA isolater (Vazyme) according to the manufacturer's instruction. 1 μg RNA sample was transcribed into cDNA with HiScript® II Q RT SuperMix for qPCR (+gDNA wiper, Vazyme), which was used as a template for quantitative mRNA levels analysis. The primers (Generay, Shanghai, China) used for PCR amplification were listed as Table 1, the whole PCR analysis was performed with ChamQ™ Universal SYBR® qPCR Master Mix kit (Vazyme) using the ABI step one plus real-time PCR system (Applied Biosystems) under the following conditions: 95 °C, 30 s; then followed by 40 cycles (95 °C, 10 s; 60 °C, 30 s); finally 95 °C, 15 s; 60 °C, 60 s; 95 °C, 15 s. All gene expression calculated by comparative Ct method was normalized to β-actin as a reference. The data was represented as a ratio relative to respective controls.

**Table 1:** Primer sequences used in the RT-PCR

Genes	Forward primer	Reverse primer
FAS	CTGAGATCCCAGCACTTCTTGA	GCCTCCGAAGCCAAATGAG
CD36	GCTTGCAACTGTCAGCACAT	GCCTTGCTGTAGCCAAGAAC
ACC	GAATCTCCTGGTGACAATGCTTATT	GGTCTTGCTGAGTTGGGTTAGCT
SREBP-1c	GGCTATTCCGTGAACATCTCCTA	ATCCAAGGGCAGTTCTTGTG
PGC-1α	TGTTCCCGATCACCATATTCC	GGTGTCTGTAGTGGCTTGATTC
MCP-1	AGGTCCCTGTCATGCTTC	GTGCTTGAGGTGGTTGTG
β-Actin	CGGTTCCGATGCCCTGAGGCTCTT	CGTCACACTTCATGATGGAATTGA

### Statistical analysis

All values were presented as mean ± S.E.M. Differences between two groups were analyzed by un-paired Student's *t*-test, and one-way analysis of variance with Dunnett's tests was used to analyze the differences among three groups by GraphPad 5.0 software (La Jolla, CA, USA). In all cases, *P* < 0.05 was regarded as a statistically significant difference.

## Results

### Notoginsenoside Fe ameliorates high-fat diet-induced obesity in C57BL/6 mice

**Fig. 1:**
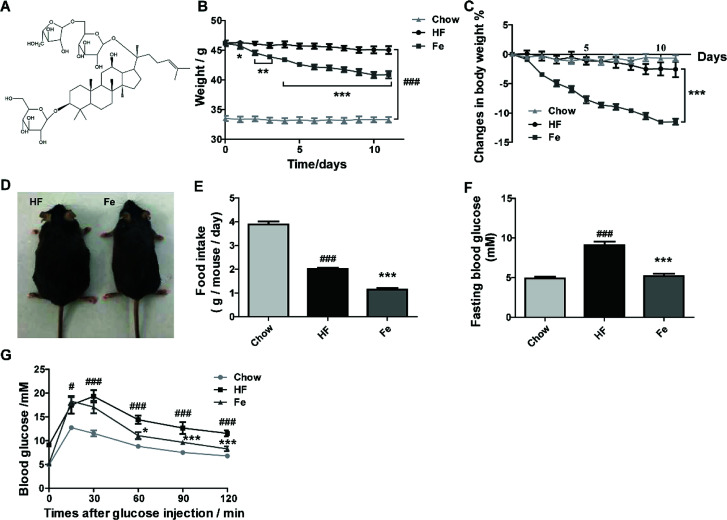
Notoginsenoside Fe ameliorates diet-induced obesity. (A) Chemical structure of notoginsenoside Fe. (B) Body weight. (C) Body weight changes. (D) Images of mice body type. (E) Food intake amount. (F) Fasting blood glucose. (G) Intraperitoneal glucose tolerance test. The blood glucose levels at the indicated intervals (15, 30, 60, 90, 120 min) after glucose injection intraperitoneally. All data were analyzed by one-way analysis of variance with Dunnett test or un-paired Student's *t*-test. *N* = 5, *P < 0.05, **P < 0.01, ***P < 0.001 *vs.* HF group; ^#^*P* < 0.05, ^###^*P* < 0.001 *vs.* chow. Notoginsenoside Fe was expressed as Fe.

To test whether notoginsenoside Fe (Fig. 1A) has effect on metabolic disorders, we used DIO mouse model. After 3 month-high-fat diet induction, body weight of C57 mice were dramatically increased compared to chow diet mice (Fig. 1B). However, the notoginsenoside Fe treatment decreased body weight of DIO mice by 12% compared with HF group (Fig. 1B and C). There was no difference of body weight change in both HF and chow group during the treatment period, suggesting that the body weight change in notoginsenoside Fe group was due to the drug effect (Fig. 1C). The somatotype of notoginsenoside Fe-treated mice was significantly diminished compared to that in HF group as shown in Fig. 1D. To address why body weight decreased in notoginsenoside Fe group, we measured food intake amount of these mice. As expected, the notoginsenoside Fe treatment decreased food intake significantly compared to the HF group (Fig. 1E), suggesting that the weight-loss effect of notoginsenoside Fe is mediated at least through reduction of feeding.

Additionally, we measured blood glucose levels in the mice. The fasting blood glucose of HF group was evidently higher than chow group, while Fe reduced fasting blood glucose compared to HF (Fig. 1F). During IPGTT, blood glucose levels in HF group were much higher than chow group at each time point (Fig. 1G). However, Fe decreased blood glucose level at the time points of 60, 90 and 120 min following glucose injection when compared with HF group (Fig. 1G). These results indicate that Fe administration ameliorates glucose tolerance in DIO mice.

To assay whether Fe reduces food intake and body weight in lean mice, chow diet-fed mice were injected with notoginsenoside Fe as the treatment of DIO mice. The results showed that there was no difference on feeding or body weight loss between saline and notoginsenoside Fe i.p injection in chow diet-fed mice, suggesting that Fe may exert specific effect on DIO mice (ESI Fig. S1A and B†). Additional, we performed C-Fos immunohistochemistry on chow-fed mice treated with saline or notoginsenoside Fe. Fe did not induce C-Fos expression either in ARC (Fig. S1C and D†) or in PVH (Fig. S1E and F†) comparing with saline group, confirming that notoginsenoside Fe showed no effect on chow mice.

### Notoginsenoside Fe treatment ameliorates hepatic steatosis and fat accumulation in diet-induced obese mice

**Fig. 2:**
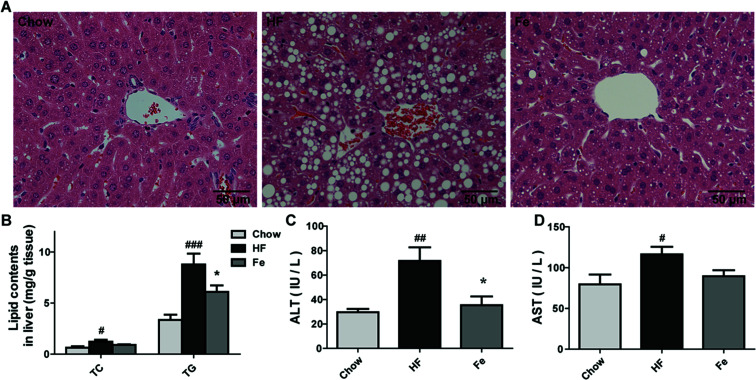
Notoginsenoside Fe treatment ameliorates hepatic steatosis in diet-induced obese mice. (A) Liver section morphology stained with H&E. (B) TC and TG contents in the liver. (C and D) The serum ALT and AST levels. Comparisons among three groups or two groups were analyzed by one-way analysis of variance with Dunnett test or un-paired Student's *t*-test. *N* = 5, *P < 0.05 *vs.* HF group; ^#^*P* < 0.05, ^#^*P* < 0.05, ^###^*P* < 0.001 *vs.* chow. Notoginsenoside Fe was expressed as Fe.

Liver is a target organ for lipid metabolism, excessive fat accumulation in the liver causes hepatic steatosis.^28^ We assessed whether notoginsenoside Fe treatment affects hepatic morphology and fat accumulation in DIO mice. In Fig. 2A, DIO mice displayed notable lipid accumulation, macrovesicular and microvesicular steatosis compared with the integral and normal hepatic structure of chow-fed mice. In contrast, treatment with notoginsenoside Fe efficiently ameliorated hepatic steatosis and tissue structure disruption. Similarly, the hepatic TC and TG levels were significantly increased in DIO mice than those in the chow mice (Fig. 2B). Notoginsenoside Fe markedly reversed the elevated TG level, suggesting that notoginsenoside Fe may improve lipid metabolism in the liver.

Serum aminotransferase levels, especially ALT and AST, are usually used as signs of damaged or inflamed liver.^29^ In our study, serum ALT and AST levels were remarkably elevated in DIO mice, especially the ALT level, which increased approximately two-fold in HF group than those in chow group (Fig. 2C and D). A significant lower level of ALT level and a trend of lower AST were seen in notoginsenoside Fe-treated group compared to that in HF group, suggesting that notoginsenoside Fe improved liver function (Fig. 2C and D). Taken together, our data suggested that notoginsenoside Fe could reduce lipid accumulation in liver and attenuate hepatic steatosis in DIO mice.

### Notoginsenoside Fe treatment reduces fat mass and lipogenesis in diet-induced obese mice

**Fig. 3:**
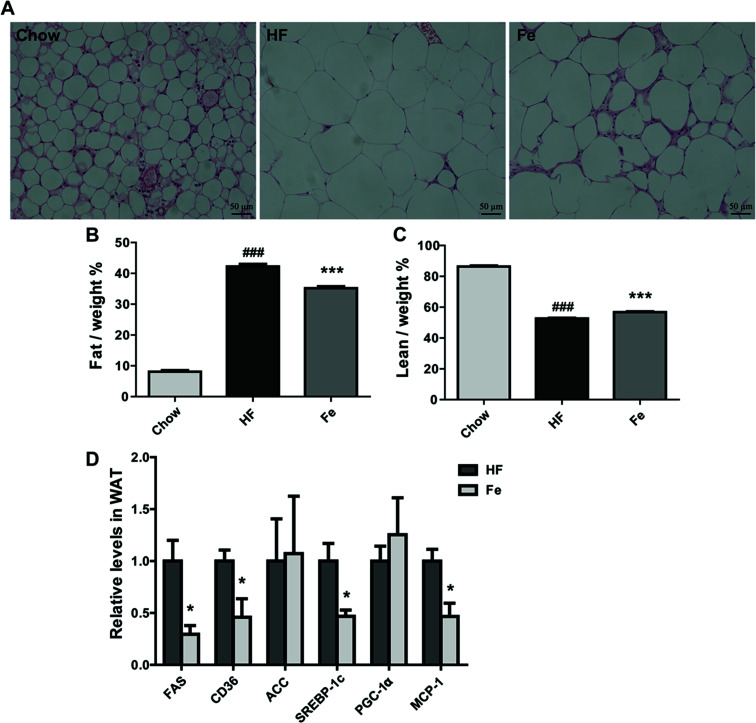
Notoginsenoside Fe treatment reduces fat mass in diet-induced obese mice. (A) WAT section morphology stained with H&E. (B and C) The percentages of fat and lean compared with body weight. (D) The mRNA expression level of genes related to glucose and lipid metabolism. All data were analyzed by one-way analysis of variance with Dunnett test or un-paired Student's *t*-test, respectively. *N* = 5, *P < 0.05, ***P < 0.001 *vs.* HF group; ^###^*P* < 0.001 *vs.* chow. Notoginsenoside Fe was expressed as Fe.

As demonstrated in Fig. 3A, the H&E pathological examination showed that adipocyte size was remarkably larger than the chow group, which was reduced in notoginsenoside Fe-treated group. Body composition information is one of important indexes to assess anti-obesity drug effect. So we tested fat mass in DIO mice. Consistent with the result above, the MRI data showed that notoginsenoside Fe remarkably reduced fat mass but not lean mass in DIO mice (Fig. 3B and C). Even, the lean/body weight increased after notoginsenoside Fe treatment (Fig. 3C), indicating that the anti-obesity effect of notoginsenoside Fe was due to reduced fat mass.

Next, we measured expression of the genes involved in lipogenesis in WAT of the DIO mice. Fig. 3D showed that fatty acid synthase (FAS), sterol regulatory element-binding protein (SREBP-1c), monocyte chemotactic protein-1 (MCP-1) and CD36 mRNA levels were notably reduced in notoginsenoside Fe-treated group compared with that in HF group. Acetyl-CoA carboxylase (ACC) and peroxisome proliferator-activated receptor gamma coactivator-1α (PGC-1α) mRNA expression levels showed no differences between HF group and Fe-treated group. The results demonstrated that notoginsenoside Fe could attenuate lipid accumulation through regulation of lipogenesis gene expression in adipocytes.

### Notoginsenoside Fe increases energy expenditure in DIO mice

**Fig. 4:**
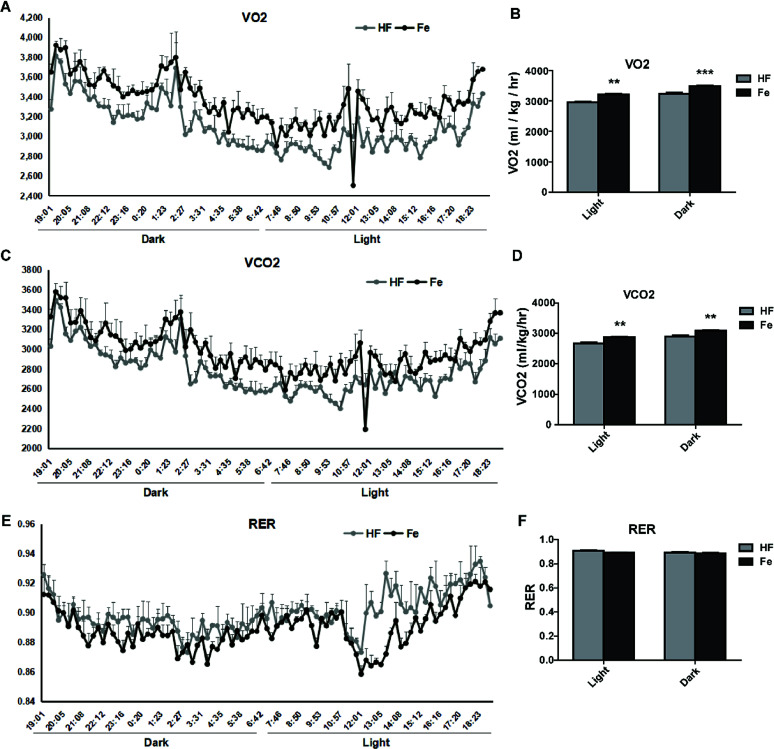
Effect of notoginsenoside Fe on metabolic measures. (A–F) VO_2_, VCO_2_, and RER values. The data represented as average of three dark and three light cycles, and were analyzed by un-paired Student's *t*-test. *N* = 5, **P < 0.01, ***P < 0.001 *vs.* HF group. Notoginsenoside Fe was expressed as Fe.

To test whether notoginsenoside Fe regulates energy expenditure, we measured VO_2_ (Fig. 4A and B) and VCO_2_ (Fig. 4C and D) and RER (Fig. 4E and F) with CLAMS. As shown in Fig. 4, notoginsenoside Fe treatment could markedly enhanced oxygen consumption and carbon dioxide emissions compared to those in HF group in both dark and light cycles, but caused no difference in RER between HF group and notoginsenoside Fe-treated group, suggesting notoginsenoside Fe increases energy expenditure in DIO mice.

### I.c.v injection of notoginsenoside Fe lowers the food intake in DIO mice

**Fig. 5:**
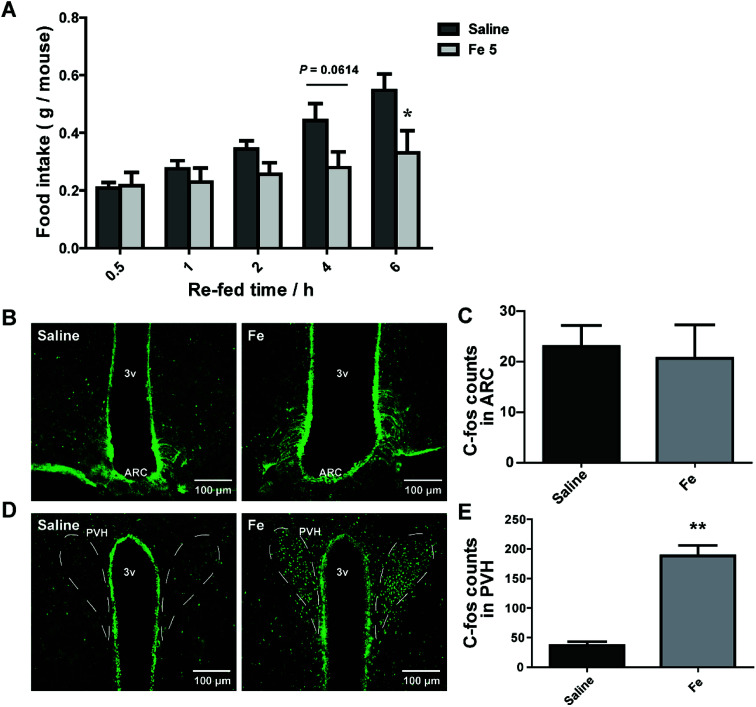
I.c.v injection of notoginsenoside Fe affects food intake of obese mice. (A) Food intake of DIO mice after i.c.v injection with saline and notoginsenoside Fe. (B–E) Notoginsenoside Fe induced C-Fos expression in the ARC and PVH of DIO mice, respectively. The data were analyzed by un-paired Student's *t*-test. *N* = 5, *P < 0.05, **P < 0.01 *vs.* saline group. Notoginsenoside Fe was expressed as Fe.

Central nervous system (CNS), especially hypothalamus contains several different types of neurons modulating food intake and energy expenditure. The brain is the hub to respond to messages from peripheral tissue for controlling feeding.^30^ There was a possibility that the inhibitory effect of notoginsenoside Fe in feeding may directly act on the brain. We thus delivered notoginsenoside Fe directly into the brain of DIO mice through guided cannula of over-night-fasted DIO mice. The results showed that notoginsenoside Fe inhibited food intake amount within 6 h after i.c.v injection compared to the DIO mice (Fig. 5A), suggesting that notoginsenoside Fe may have a direct role in the hypothalamus in feeding control.

To investigate whether notoginsenoside Fe i.c.v injection could alter neuron activity in the hypothalamus, we carried out C-Fos immunohistochemistry on brain slices of DIO mice after i.c.v injection of saline or notoginsenoside Fe. C-Fos expression was generally regarded as a marker for neuron activity levels. Fig. 5B and C showed that there was no difference of C-Fos expression between saline and notoginsenoside Fe-treated group in ARC. However, notoginsenoside Fe i.c.v injection induced remarkably higher C-Fos expression in hypothalamic PVH compared to saline group (Fig. 5D and E). These results raise the possibility that notoginsenoside Fe directly activate PVH neurons to regulate food intake in DIO mice.

## Discussion

Here, we showed that a component of *Panax notoginseng* leaves, notoginsenoside Fe could remarkably reduce food intake in HF diet-induced obesity mice. Additional, notoginsenoside Fe improved glucose tolerance (IPGTT), protected liver structure integrity and function, decreased liver TG content, and inhibited the lipogenesis-related genes expression in WAT of DIO mice.

Suppression of food intake is a major current therapeutic strategy for obesity. Our data demonstrated that notoginsenoside Fe induced neuron activity in the hypothalamus. The hypothalamus is a vital integrative center for peripheral and central signals controlling food intake and energy expenditure.^31^ ARC is a primary target of metabolic and hormonal signals related to energy homeostasis from the periphery.^32^ Two major types of neurons agouti gene-related protein/neuropeptide Y (AgRP/NPY) and proopiomelanocortin (POMC) locates in the ARC regulate food intake, through projections to PVH and other brain areas. PVH serves as an important integrator for signals regulating food intake and energy expenditure.^32–34^ Previous studies demonstrated that PVH lesion caused hyperphagia while activation of these neurons reduces food intake.^35^ Interestingly, in our study, i.c.v notoginsenoside Fe injection specifically induced abundant C-Fos expression only in the PVH but not in the ARC, suggesting that notoginsenoside Fe may target a special group of neuron in the PVH to reduce food intake. PVH contains different types of neurons known to play a role in feeding.^6,9,36^ Moreover, we performed i.p injection of notoginsenoside Fe in chow group of mice and the results exhibited that i.p treatment of notoginsenoside Fe caused no difference in food intake, body weight or C-Fos expression in PVH neurons in chow diet-fed mice compared to saline group (ESI Fig. S1†), suggesting that notoginsenoside Fe has specific effect on DIO mice. Collectively, further studies are required to narrow down which group of PVH neurons that mediate the notoginsenoside Fe action on reducing food intake of DIO mice.

In addition to food intake effects, notoginsenoside Fe also increased energy expenditure. Since obesity results from chronic imbalance between calorie intake and energy expenditure, reducing body weight can also be achieved through increasing energy expenditure. Our CLAMS data showed that the notoginsenoside Fe-treated mice increased O_2_ consumption and CO_2_ production, suggesting that notoginsenoside Fe increases energy expenditure. The adipose tissue participates in the regulation of lipid homeostasis and energy balance by storing energy in the form of TG.^37,38^ SREBPs have been established as lipid synthetic transcription factors involved in fatty acids uptake, biosynthesis and metabolism through its downstream gene expression related to *de novo* lipogenesis, such as ACC, FAS, and fatty acid transport protein CD36.^39–41^ Recent studies reported that decreased SREBP-1c could inhibit lipogenesis.^42–44^ In our results, notoginsenoside Fe treatment reduced the expression of SREBP-1c and those of its target genes FAS and CD36 in WAT of DIO mice, suggesting that notoginsenoside Fe could inhibit lipogenesis and fatty acid synthesis in WAT to reduce lipid accumulation.

In conclusion, notoginsenoside Fe could alleviate HF diet-induce obesity and other metabolic defects through reduction of food intake, increase of energy expenditure, and inhibition of lipogenesis through both peripheral and central mechanisms. These findings suggested that notoginsenoside Fe could be used as a potential natural product for the treatment of obesity.

## Conflicts of interest

All authors declare that they are no conflict of interest.

## Supplementary Material

RA-009-C8RA07842D-s001
