# Supplementary material for: Notoginsenoside Fe suppresses diet induced obesity and activates paraventricular hypothalamic neurons
Source: RSC Adv. 2019 Jan 11;9(3):1290–8. doi: 10.1039/c8ra07842d (PMC9059641; doi:10.1039/c8ra07842d)

Electronic Supplementary Material (ESI) for RSC Advances.
This journal is © The Royal Society of Chemistry 2018

Supplementary Fig. S1 *I.p* injection with notoginsenoside Fe have no effect on body weight and feeding in chow mice. (A) Body weight. (B) Food intake. (C-D) C-Fos expression in ARC. (E-F) C-Fos expression in PVH.

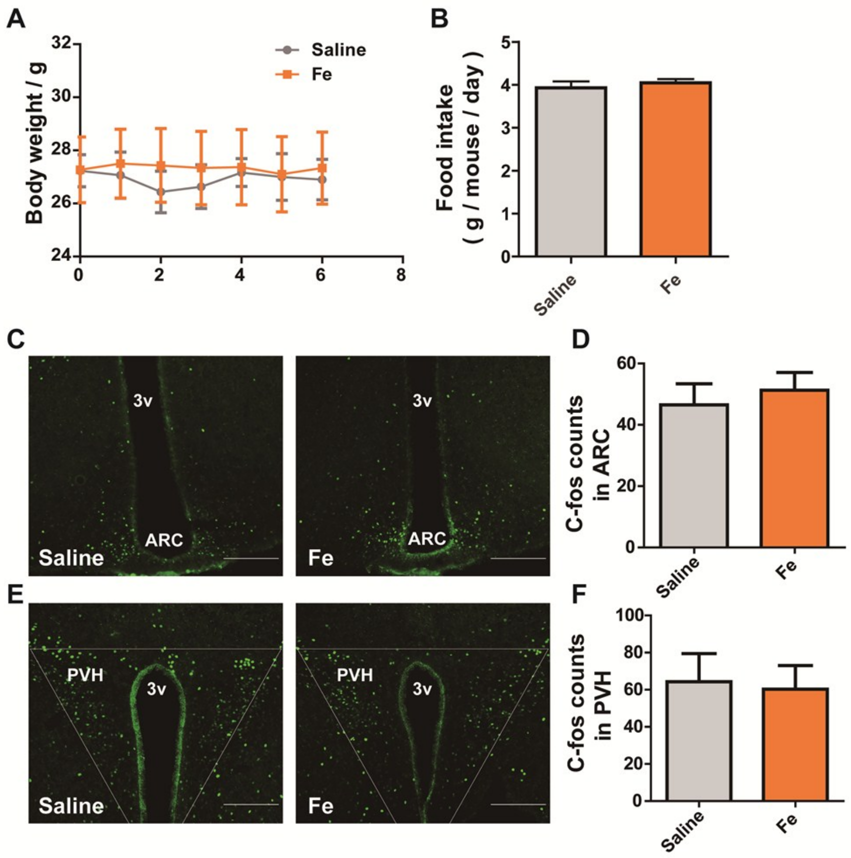

Supplement: RA-009-C8RA07842D-s001 [file RA-009-C8RA07842D-s001.pdf]
